# Die somatische Dysfunktion der Halswirbelsäule und ihr komplexes klinisches Bild: Grundlagen der manualmedizinischen Diagnostik von Zervikobrachialgie und zervikozephalem Syndrom

**DOI:** 10.1007/s00132-022-04228-7

**Published:** 2022-03-03

**Authors:** Florian Max-Josef Wagner

**Affiliations:** Starnberger Wiese 44, 82319 Starnberg, Deutschland

**Keywords:** Zervikotrigeminale Konvergenz, WDR, Propriozeptiver Reiz, Manuelle Medizin, Blockierung, Trigeminocervical interactions, WDR, Proprioceptive input, Manual medicin, AAO-complex

## Abstract

Die segmentale und somatische Dysfunktion der Halswirbelsäule kann aufgrund komplexer funktionell-anatomischer und neurophysiologischer Verschaltungen dem Untersucher einen variantenreichen, aber teils schwer nachvollziehbaren Symptomkomplex bieten. Auf Grundlage von zervikotrigeminalen und intersegmentalen Konvergenzen im Bereich des Rückenmarks der Halswirbelsäule und des Hirnstammes lassen sich diese Symptome, die als zervikozephales Syndrom und Zervikobrachialgie zusammengefasst werden, nachvollziehen. Eine fundierte manualmedizinische Untersuchung kann, nach Ausschluss bedrohlicher Differenzialdiagnosen, das Vorliegen einer Blockierung der Halswirbelsäule als mögliche Ursache oder als Teilaspekt der Beschwerden demaskieren. Eine manualmedizinische Behandlung mit dem Setzen gezielter antinozizeptiver propriozeptiver Reize ist dann eine sinnvolle therapeutische Option.

## Abstract (en)

Patients suffering from a segmental and somatic dysfunction of the cervical spine often present a wide variety of clinical symptoms related to cervicobrachial or cervicocephalic syndrome. These symptoms might evolve out of complex neural intersegmental or trigeminocervical interactions in the brain stem or the spinal cord of the cervical spine. After the exclusion of life-threatening preconditions, a careful physical examination with aspects of manual medicine aspects might unmask the cervical dysfunction as the primary cause of the symptoms. Treatment with manual medicine on the basis of a segmental antinociceptive proprioceptive input might then be an appropriate therapeutic approach.

Kein anderer Wirbelsäulenabschnitt kann im Falle einer segmentalen und somatischen Dysfunktion (Blockierung) aufgrund seiner komplexen neurophysiologischen, neuroanatomischen und biomechanischen Verschaltungen ein so vielfältiges, teils dramatisch imponierendes klinisches Spektrum an Symptomen bieten, wie die HWS. Wegen der Komplexität, aus Sorge vor unzureichender diagnostischer Abklärung, dem Übersehen einer bedrohlichen Differenzialdiagnose oder Angst vor Schädigung bei inadäquater Behandlungstechnik wird die Halswirbelsäule häufig gar nicht oder nur unzureichend manualmedizinisch behandelt.

## Einleitung

Die segmentale und somatische Dysfunktion der Halswirbelsäule und ihr klinisches Spektrum, von der Zervikobrachialgie bis zum zervikozephalen Syndrom, ist unverändert für viele manualmedizinisch tätige Ärzte eine Herausforderung. Unverändert ist auch die Skepsis vieler Fachgesellschaften bezüglich der Indikation einer manualmedizinischen Therapie. Infrage gestellt wird aber auch das vor allem auf neurophysiologischen Grundlagen basierende Erklärungsmodell, warum manuelle Medizin auch und besonders in diesem Bereich eine herausragende therapeutische Option sein kann [1]. Dabei zeigt sich hier, mit den Erkenntnissen translationaler Forschung untermauert [2], ein schlüssiges manualmedizinisch diagnostisches wie therapeutisches Konzept, das bei genauer Betrachtung wenig Raum für Zweifel lässt [3].

Die Kombination verschiedenster Symptome, die auf den ersten Blick verschiedenen Fachgebieten wie Neurologie, der HNO- oder Augenheilkunde zugeordnet werden, kann Ausdruck ein und derselben grundlegenden Problematik sein: der gestörten Verarbeitung nozizeptiver segmentaler Afferenzen aus der Halswirbelsäule und deren Einfluss auf die an der zervikotrigeminalen oder intersegmentalen Konvergenzreaktion beteiligten Strukturen. Nach bedarfsweisem interdisziplinärem Ausschluss bedrohlicher Differenzialdiagnosen sollte die manualmedizinische Diagnostik mit ihrem klaren 3‑Schritte-Algorithmus, der segmentalen Mobilitätsprüfung (M), der Irritationspunktsuche (I) und der Provokation des Irritationspunktes (P), ein integraler Bestandteil des weiteren Untersuchungsganges sein.

Ein sicher und effektiv umsetzbarer, klarer diagnostischer und therapeutischer Algorithmus ist im klinischen Alltag hilfreich und kann dem nur scheinbaren „Symptommonster“ der HWS-Blockierung die Bedrohlichkeit nehmen. Dafür ist es sinnvoll, die HWS vor allem nach neurophysiologischen Aspekten in 2 Bereiche zu unterteilen:

- die obere HWS (C0/C1–C2/C3) mit dem Symptomkomplex des zervikozephalen Syndroms auf Basis der zervikotrigeminalen Konvergenz,
- die untere HWS mit zervikothorakalem Übergang (C3/C4 bis ca. C7/Th1) und dem pseudoradikulären Schmerzbild der Zervikobrachialgie („Nacken-Schulter-Arm-Syndrom“).

Die Möglichkeit, manualmedizinisch in das funktionelle Blockierungsgeschehen der HWS durch Setzen gezielter propriozeptiver Reize einzugreifen und damit auch komplexe Beschwerdebilder zu behandeln, erklärt sich aus Kenntnis der im Folgenden erklärten neuroreflektorischen Prozesse, der Muskelphysiologie wie auch der grundlegenden Anatomie und Biomechanik. Therapeutisch werden neben den atraumatischen manipulativen Techniken vermehrt auch segmentale mobilisierende Techniken, neuromuskuläre Techniken, Positionierungs- und Druckpunkttechniken sowie deren sequenzielle Kombination eingesetzt.

Durch Setzen gezielter propriozeptiver Reize wird in das Blockierungsgeschehen an der HWS eingegriffen

Ein sicherer Umgang in diesem Beschwerdegebiet findet reichlich Anwendungsmöglichkeiten bei zunehmenden, vor allem haltungsbedingten, funktionellen Störungen der HWS auch bei jüngeren Patienten durch z. B. sitzende Bildschirmarbeit („Späherhaltung im Homeoffice“) sowie Freizeitgestaltung in vergleichbarer Haltung und Umgebung. Patienten mit zumeist etwas „undankbaren“ Symptomen wie Gleichgewichtsstörung, Ohrgeräusch, Kopfschmerz oder Schulter-Arm-Syndrom in Verbindung mit einem Blockierungsgeschehen der HWS kann nach entsprechender multidisziplinärer Abklärung mithilfe eines manualmedizinischen Ansatzes eine sinnvolle therapeutische Option angeboten werden.

## Fallbeispiel: Zervikozephales Syndrom

### Anamnese

Eine 38-jährige Patientin, selbstständig als Kosmetikerin und medizinische Fußpflegerin, berichtet über seit vielen Monaten wiederkehrende Nacken- und Spannungskopfschmerzen, die sie auf die Zwangshaltung v. a. bei der Fußpflege zurückführt. Die Beschwerden verstärkten sich regelmäßig entsprechend der täglichen Arbeitsdauer und subjektiv empfundener Stressbelastung (finanzielle Einbußen während Corona-Lockdown). Nach Hausarbeit am Wochenende mit Überkopfarbeit und schwerem Heben, kam es zu einer massiven Verschlechterung der bekannten Schmerzsymptome, zusätzlich stechenden Ohrschmerzen mit summendem Ohrgeräusch links, retrobulbärem Stechen beidseits, reduzierter Kieferöffnung und Übelkeit. Nichtsteroidale Antirheumatika (Ibuprofen 600 mg 1‑1-1) brachten keine Linderung. In den folgenden Tagen traten Gleichgewichtsstörungen und Konzentrationsstörungen mit Angstattacken auf. Nach einmaligem Kollaps erfolgte die Einweisung ins Krankenhaus zur stationären Abklärung. Die Gesamtschau der multidisziplinären Abklärung (labormedizinisch, kardiovaskulär, neurologisch, HNO-ärztlich, radiologisch [Röntgen HWS, MRT, s. unten]) brachte keine richtungsweisende Diagnose, sodass die Patientin bei leicht rückläufiger Intensität des Gesamtbefundes entlassen wurde.

### Diagnostik

#### Klinischer Untersuchungsbefund.

Hirnnervenstatus, Motorik und Reflexstatus im Versorgungsgebiet der HWS unauffällig. Unterberger-Tretversuch unsicher ohne eindeutiges Seitabweichen. Deutlicher muskulärer Hartspann der rechten paravertebralen Halsmuskulatur. Bei gehaltener HWS-Flexion schmerzhafte Rotationseinschränkung nach links (ROM bei HWS-Flexion: Rot. R/L: 40°/0/20°), hierbei auch verstärktes retrobulbäres Stechen. Seitneigung links reduziert (SB R/L: 30°/0/20°). Reklination endgradig schmerzhaft. Linksrotationsempfindlicher Irritationspunkt am HWK 2 rechts (C2+ li).

#### Röntgen der HWS.

Steilstellung der HWS, unauffälliger altersentsprechender Befund.

#### MRT der HWS.

Streckfehlstellung der HWS, keine pathologischen Veränderungen an der gesamten HWS.

#### Diagnose.

Zervikozephales Syndrom mit Gleichgewichtsstörung und Konzentrationsstörung (Dizziness), Kopfschmerzen (Dermatom C2) und psychovegetativer Dysregulation. Segmentale und somatische Dysfunktion der oberen HWS. Schmerzexazerbation nach Zwangshaltung in Reklination (Überkopfarbeiten) bei chronischer statischer Fehlbelastung (Arbeit in Zwangshaltung) und psychosozialer Belastung („Geldsorgen“).

### Therapie

**Figure 1:**
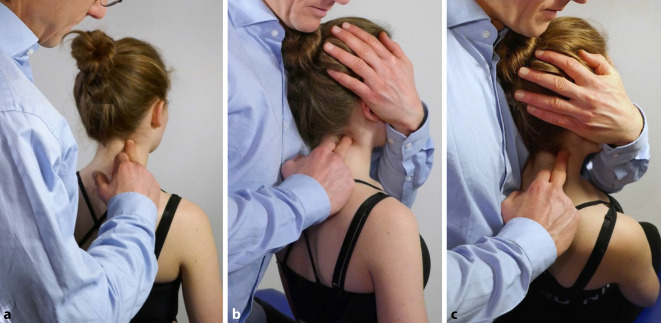

Es erfolgte die zurückhaltende Mobilisation der HWS, kombiniert mit neuromuskulären Techniken. Anschließend Druckpunktbehandlung an HWK 2 rechts mit schmerzfreier Positionierung des HWK 2 durch Rechtsrotation, Rechtsseitneigung und moderater Traktion in Kombination mit reziproker neuromuskulärer Hemmung (Abb. 1). Daraufhin deutliche Reduktion des muskulären Hartspans. 2 Tage später Re-Check, dabei schon Besserung der Rotationseinschränkung der HWS. Erneuter vergleichbarer Therapiealgorithmus. Anleitung zur Eigenbeübung mit dem Ziel der muskulären Stabilisierung der HWS, sowie Optimierung der Haltung am Arbeitsplatz. 14 Tage später Kontrolle mit > 80 % Besserung des Beschwerdebildes. Seitengleiche ROM der HWS mit nur noch leicht endgradigem Schmerzempfinden bei Rotation.

## Die obere Halswirbelsäule: zervikotrigeminale Konvergenz und zervikozephales Syndrom

Anatomisch werden nur das Os okzipitale (C0), der Atlas (C1) und der Axis (C2) zur oberen HWS gezählt. Deren Hauptbewegungen sind, neben nur geringen endgradigen passiven Bewegungen, das Nicken (Inklination/Reklination von C0/C1) und die Rotation (C0/C1, v. a. C1/C2) (Tab. 1).

**Table 1:** 

Segment	ROM (in °)	Segmentnerv	Wichtige periphere Nerven	Sensible/sensorische Besonderheiten, Dermatome	Motorisch R. dorsalis	Reflexkennmuskeln/Besonderheiten
C0/C1	Ink/Rek: 24–30 R pass: < 5 SN: 5–10	C1	N. suboccipitalis, Ansa cervicalis, regional wichtig Neuroforamina der Hirnbasis v. HN IX–XII	Dura mater im Bereich des Foramen magnum (Afferenzen häufig via Anastomose zu C2 [N occ. major])	–	Kurze Nackenmuskeln: M. rectus capitis posterior maj. et min., M. obliquus capitis sup. et inf
C1/C2	R: 38–43/Seite SN: ~ 6 F/E: 22	C2	N. occipitalis major (mit C3), Ansa cervicalis	Okzipitalregion, dorsale Schädelgrube	ATM (IP/Ins)	–
C2/C3	F/E: 8–12 SN: 10/Seite R: 9/S	C3	N. occipitalis minor, Ansa cervicalis, N. occ. tertius, N. phrenicus	Nackenregion, Facetten C2–C4, C3/C4 (Übergangssegment zwischen oHWS/uHWS)	ATM (IP/Ins)	*Zwerchfell*
C3/C4	F/E: 12–17 SN: 11/Seite R: 10/S	C4	N. phrenicus (Plex. Brachialis)	Facetten C3–C5 „Schal“-Dermatom	ATM (IP/Ins)	*Zwerchfell *(Trapeziusreflex)
C4/C5	Wie C3/C4	C5	Plex. brachialis, N. radialis (C5-Th1), N. musculocut. (C5-7), N. axillaris (C5-6), (N. phrenicus)	Kein Rumpfdermatom, Schulter (M. deltoideus)	ATM (IP/Ins)	Zwerchfell, M. deltoideus, *M. biceps brachii (Bizepsreflex)*
C5/C6	F/E: 17–21 SN: 8/Seite R: 10/S	C6	Plex. brachialis, N. radialis (C5-Th1), N. musculocut. (C5-7), N. axillaris (C5-6), N. medianus (C6-Th1)	Kein Rumpfdermatom, radialer Unterarm bis Daumen	ATM (IP/Ins)	*M. biceps brachii (Bizepsreflex), *M. brachioradialis
C6/C7	F/E: 16. 21 SN: 7/Seite R: 9/S	C7	Plex. Brachialis *N. radialis (C5-Th1)* N. musculocut. (C5-7) N. medianus (C6-Th1)	Kein Rumpfdermatom, Unterarm bis Dig. II–IV	ATM (IP/Ins)	*M. triceps brachii* *Trizepsreflex* M. pectoralis major M. pronator teres
C7/Th1	F/E: 9 SN: 4/Seite R: 8/S	C8	Plex. brachialis, N. radialis (C5-Th1), N. medianus (C6-Th1), N. ulnaris (C8, Th1)	Kein Rumpfdermatom, Unterarm bis Dig. V	ATM (IP/Ins)	*Kleinfingerballenmuskel, Trömner-Reflex*
Th1/Th2	F/E: 4 SN: 5/Seite R: 9/S	Th1	Plex. brachialis, N. radialis (C5-Th1), N. medianus (C6-Th1), *N. ulnaris (C8, Th1)*	(Kein Rumpfdermatom), ulnarseitig Unterarm und Ellbogen, symp. Grenzstrang (Horner-Syndrom)	ATM Interkostalmuskulatur	M. flexor carpi ulnaris, Hypothenar

*R* Rotation, *F* Flexion, *E* Extension, *Ink* Inklination, *Rek* Reklination, *SN* Seitneigung, *pas.* passiv, *akt* aktiv, *ATM* autochthone Muskulatur, *IP* Irritationspunkte, *Ins* Insertionspunkte, *ROM* „range of motion“, *HN* Hirnnerv (mod. aus [4–8])

Von muskulärer Seite betrachtet spielen die vor allem von dem motorischen Segmentnerv C1 innervierten kurzen Nackenmuskeln eine herausragende Rolle. Die Mm. rectus capitis posterior major und minor sowie die Mm. obliquus capitis superior und inferior sind mit tandemartig angeordnete Muskelspindeln, in einer Dichte wie sonst nur in der Augenmuskulatur [4], durchbaut. Diese dienen vor allem auch der Propriozeption, nicht nur der Bewegungsausführung und vermitteln über den sensiblen Anteil des Segmentnerv C2 Information über die Stellung des Kopfes zum Körper [5]. Aus diesem Grund kann die obere HWS neben dem optischen und vestibulären System als integraler Bestandteil der an der Raumwahrnehmung beteiligten Sinnesorgane bezeichnet werden. Von Hassenstein wurde dies in der Formel (W = Wahrnehmung der Raumlage) zusammengefasst [5]:

$$W_{\left(\text{Rumpf}\right)}=W_{\left(\text{Kopf}\right)}-W_{\left(\text{Winkel}\frac{\text{Kopf}}{\text{Rumpf}}\right)}$$

Neuroanatomisch betrachtet sollten aufgrund der sensiblen Versorgung der C3-Facetten durch den Segmentnerv C3 auch das Segment C2/C3 und das Übergangssegment C3/4 hinzugerechnet werden. Deren biomechanisches Plus ist die Seitneigung, als gekoppelte Bewegung von Flexion/Extension und Rotation (Tab. 1). Der Verlauf der A. vertebralis, mit ihrer nach dorsal gerichteten Atlasschleife, bevor sie durch die Membrana atlantooccipitalis und die Dura mater das Foramen magnum erreicht, erklärt potenzielle Zug- oder Kompressionsbelastungen auf das Gefäß bei bestimmten Kopfgelenksstellungen. Symptome einer Vertebralisschädigung sind immer ein Notfall und müssen als solche leitlinienkonform abgeklärt werden ([9, 10]; Tab. 2).

**Table 2:** 

Symptome:
Nackenkopfschmerz (50 %)
Kopfschmerz okzipital, meist auf der Seite der Dissektion (66 %) aber auch bilateral
Kopfschmerz pulsierend oder dauerhaft stechend
Langsame Steigerung der Intensität
Differenzierbar von vorbestehenden Kopfschmerzen (Migräne)
Häufig ohne Zeichen der muskulären Verhärtung oder Bewegungseinschränkung
Zerebrale Ischämien (80 %) häufig mit Wallenberg-Syndrom (ipsilaterales Horner-Syndrom, Gaumensegelparese, Hemiataxie, dissoziierte Empfindungsstörung)

Interessant sind die Positionen der Neuroforamina der Hirnnerven im Bereich der Schädelbasis und ihre anatomische Nähe zum Atlantookzipitalgelenk. Durch das ventrolateral der Okziputkondylen und dem Foramen magnum gelegene Foramen jugulare ziehen die HN IX, X, XI. Der Canalis hypoglossus und in ihm sein namensgebender Hirnnerv XII „tunneln“ sogar die Okziputkondylen.

**Figure 2:**
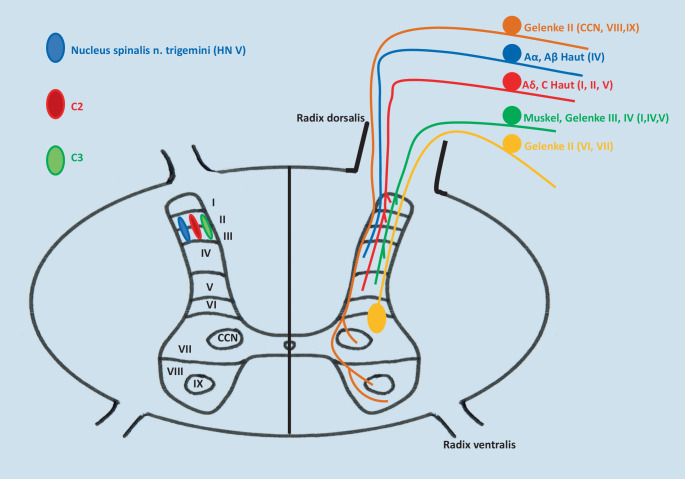

Komplex, aber auch besonders diagnostisch hilfreich, sind die neurophysiologischen Zusammenhänge im Bereich des Hirnstamms (Mittelhirn, Pons, Medulla oblongata), die unter dem Schlagwort der „zervikotrigeminalen Konvergenz“ zusammengefasst werden. Beteiligt an der Konvergenz am Nucleus spinalis n. trigemini sind neben Afferenzen aus C1 bis C3, das Zerebellum, die Formatio reticularis, der Thalamus (Nucleus ventralis posteromedialis) sowie prothopathische (Schmerz, Temperatur, grober Druck) und propriozeptive Afferenzen aus den im Hirnstamm gelegenen Hirnnervenkerne V, VII, IX, X, XI, XII, eng verschaltet mit den HN-Kernen VIII und III. Der Nucleus spinalis n. trigemini übernimmt dabei die Funktion des WDR-Neuronenpools des kaudaleren Rückenmarks und wird als „medulläres Hinterhorn“ bezeichnet. Im schematischen Querschnitt des Rückenmarks auf Höhe C2 ([5]; Abb. 2) sind die engen Lagebeziehungen der einzelnen neuronalen Strukturen deutlich erkennbar.

**Figure 3:**
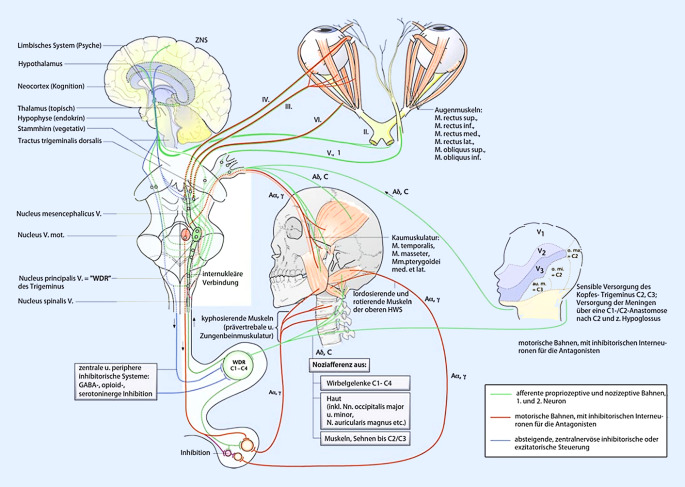

Mit dem Wissen um die reichhaltigen Verschaltungen der vielen unterschiedlichen somatosensiblen, viszerosensiblen und sensorischen Afferenzen, die an der zervikotrigeminalen Konvergenzreaktion beteiligt sein können, erklärt sich auch der bunte klinische Symptomkomplex, der bei einer Dysfunktion im Bereich der oberen HWS, die klinisch als „zervikozephales Syndrom“ bezeichnet wird, entstehen kann (Abb. 3).

Diagnostisch hilfreich sind die komplexen neurophysiologischen Zusammenhänge im Bereich des Hirnstamms

Die einzelnen Symptome, die in verschiedenster Ausprägung an dem Vollbild des zervikozephalen Syndroms beteiligt sein können (Tab. 3), werden der Übersicht halber systematisch anhand ihrer neurophysiologischen und anatomischen Bezüge dargestellt:

**Table 3:** 

Symptome:
Kopfschmerzen (Zervikozephalgie)
Gesichts‑, Schulter‑, Nacken‑, Rückenschmerzen
Gleichgewichtsstörungen (zervikozephaler „Schwindel“, besser Dizziness)
Unspezifische Augensymptome (Fokussierstörung, Schlierensehen)
Unspezifische Ohrsymptome (Ohrgeräusch, Tinnitus, Hall)
Globusgefühl, Dysphonie, Räusperzwang, „Schluckauf“
Vegetative Störungen (Schwitzen, Übelkeit, Palpitationen, Blutdruckschwankungen)
Stimmungsauffälligkeiten (z. B. Angst, Trauer, „Stress“)
Konzentrationsstörungen
Motorische, koordinative Störung (z. B. gestörtes Schriftbild, Kiefergelenksdysfunktion)

### Visuelles System (HN II, III, IV, VI).

Das optische System zählt neben dem propriozeptiven und vestibulären System zu den drei eng miteinander kommunizierenden gleichgewichtsregulierenden Systemen. Funktionsstörungen der Augenmuskeln können zu zeitweisem Auftreten von Doppelbildern oder Augenflimmern führen. Dies kann durch eine ständige und somit erschöpfende Fusionsmehrarbeit Konzentrationsstörungen und Kopfschmerzen verursachen. Die Augenmuskulatur ist vor allem mit der C1–C3 innervierten Halsmuskulatur funktionell eng verzahnt, was sich durch den Nick-Lese-Versuch nach Hassenstein eindrücklich zeigen lässt [5]. Diese enge Verzahnung erklärt, warum nozizeptive Afferenzen aus einer dysfunktionalen Halsmuskulatur zu Sehstörungen und vice versa (Heterophorie) führen können [13]. Diese enge Verbindung kann auch die Wirkungsweise der Blickfazilitierung im Rahmen einer manualmedizinischen Therapie erklären. Über den medialen Vestibulariskern besteht die Verknüpfung des visuellen mit dem Gleichgewichtssystem (siehe vestibulokochleäres System). Über den aufsteigenden Teil des Fasciculus longitudinalis medialis werden sowohl Afferenzen als auch Efferenzen zwischen allen vestibulären Kerngruppen und allen Kernen der Okulomotorik projiziert [5].

### Sensomotorik (HN V, VII, IX, XI, XII).

Propriozeptive Afferenzen des Kiefers, der Zähne und der meisten Kaumuskeln erreichen zunächst den mesenzephalen Trigeminuskern. Protopathische Afferenzen (Schmerz, Temperatur, grober Druck) projizieren zum spinalen Trigeminuskern (Nucleus spinalis n. trigemini), der somatotop in drei Zonen gegliedert. Bei Störungen in diesem zentralen Kernbereich kommt es zu ringförmigen Sensibilitätsstörungen (Sölder-Linien) im Vergleich zu den drei kraniokaudal gegliederten Befundregionen bei der peripheren Nervenläsion (HN V/1 – HN V/3). Im spinalen Trigeminuskerngebiet konvergieren unter anderem auch dünnkalibrige Afferenzen der oberen HWS (C1–C3). Dies ist besonders wichtig für unsere manualmedizinische Betrachtung, da sich aus dieser Konvergenz die Interaktionen der HWS-Dysfunktion und der übertragenen Schmerzen im Versorgungsgebiet des N. trigeminus (HN V) verstehen lassen. Der N. facialis (HN VII) verhält sich analog dem N. mandibularis (HN V/2). Er ist an der motorischen Versorgung des Mundbodens inklusive des M. digastricus venter posterior beteiligt. Der N. glossopharyngeus (HN IX) versorgt sensibel den Rachenraum, sensorisch den hinteren Zungenbereich und motorisch das Gaumensegel. Der N. accessorius (HN XI) innerviert den M. sternocleidomastoideus und M. trapezius pars descendens, über den myofasziale Verbindungen bis zur tiefen LWS bestehen. Der rein somatoefferente N. hypoglossus (HN XII), als Hauptnerv der Zungenmotorik auch „Radix anterior von C0“ genannt [8], verfügt über eine ganze Reihe „geliehener“ afferenter Nervenfasern, vorwiegend der Segmentnerven C2 und C3 [5], mit denen er über die Ansa cervicalis auch die untere Zungenbeinmuskulatur (Mundöffnung, HWS-Flexion) innerviert. Besonders die tiefsomatischen Afferenzen der Zunge über die HN V3, IX und X, werden ohne Umweg über die Medulla oblongata direkt auf der Ebene der oberen zervikalen Segmente über die Hinterhorn-WDR-Neurone verschaltet. Damit besteht eine direkte, unmittelbare Verbindung der Zunge zu der motorischen Steuerung der HWS über die Axonkollateralen der zervikalen Segmente. Diese anatomische Nähe kann therapeutisch zur Zungenfazilitation bei manualmedizinischer Behandlung genutzt werden, bei der vor allem die Seitbewegung der Zunge in der Mundhöhle mit der Bewegung der HWS funktionell zusammenwirken kann. Auch kann neuroanatomisch nachvollzogen werden, warum Patienten bei Blockierungen der oberen HWS häufig ein Globusgefühl oder eine „Glossitis“ beklagen. Dermatome der HN V, VII, IX und X finden sich an der Ohrmuschel und im äußeren Gehörgang [8].

### Vestibulokochleäres System (HN VIII).

Der N. vestibulocochlearis als Doppelnerv ist für die Sinnesqualitäten Gleichgewicht und Hören zuständig. Er besitzt zwei Kernkomplexe: vier Vestibulariskerne und zwei Kochleariskerne, mit vielfältigen Verschaltungen zu fast allen anderen Stammhirnkernen und dem Kleinhirn [14]. Die anterograd und retrograd nachgewiesenen anatomischen Projektionen von den Trigeminusfasern und der vorwiegend tiefsomatischen C1/C2-Afferenzen zu beiden Kochleariskernen werden als mögliche Ursachen von Tinnitus und diversen anderen Hörstörungen bei gestörter zervikotrigeminaler Konvergenz auf Basis einer Dysfunktion der Segmente C0–C3 diskutiert [15].

Der Vestibulariskernkomplex wird ipsilateral direkt von spinalen Afferenzen erreicht, als auch indirekt kontralateral über Projektionen aus dem für zervikale Muskelafferenzen wichtigen Nucleus cervicalis centralis. Vor allem sind es hier spinale Afferenzen von C0–C3, die eine Rolle spielen.

Die bei einer Blockierung der oberen HWS gestörte Wahrnehmung der Kopf-Körper-Stellung, vermittelt durch die intensiv mit Muskelspindeln ausgestatteten kurzen Nackenmuskeln („Nackensensoren“), kann nicht durch die Leistung der anderen beteiligten visuellen und vestibulären Systeme ausgeglichen werden. Über die direkte Konvergenz von vestibulären und gestörten spinalen Afferenzen, den Verschaltungen der Vestibulariskerne als zentrale Koordinationsstelle von Augen‑, Kopf- und Körperstellung sowie den thalamischen und kortikalen Projektionen zum Bewusstwerden der Lage im Raum, kann es zu Bewegungsunsicherheiten bis hin zu Gleichgewichtsstörung (Dizziness) kommen [5, 16]. Typischerweise fehlt dieser „hochzervikalen“ Form der Gleichgewichtsstörung meist die Komponente Drehschwindel und Horizontalnystagmus [17]. Eine neuaufgetretene Gleichgewichtsstörung sollte aber aufgrund der manchmal schwer einzuordnenden Differenzialdiagnosen in jedem Fall neurologisch oder HNO-ärztlich abgeklärt werden.

### Viszerovegetatives System (v. a. HN X).

Der N. vagus beinhaltet nicht nur parasympathische Efferenzen, sondern mit dem R. meningeus auch afferente Fasern aus der hinteren Schädelgrube („Kopfschmerz“) und über den R. auricularis auch Fasern aus seinem „Dermatom“, dem äußeren Gehörgang („Ohrenschmerz“). Er führt sowohl sensible (Berührung/Temperatur) als auch geschmackssensorische Afferenzen aus der Zunge. Allgemeine Viszeroafferenzen vermitteln unter anderem Informationen aus Brust und Baucheingeweiden (bis linke Kolonflexur), von Druckrezeptoren im Aortenbogen und Chemorezeptoren im Glomus aorticum [8]. Irritierende Konvergenzen auf seine autonomen Funktionen führen zu Übelkeit, Magen-Darm-Beschwerden, zu Störungen des Bronchialsystems und des Herzens. Dabei wird er heute nicht mehr als der Antagonist des sympathischen Grenzstrangs gesehen, sondern als Synergist in der komplexen Steuerung autonomer Funktionen. Seine Afferenzen haben einen großen Einfluss auf das limbische System sowie den Thalamus mit Projektionen zum Inselkortex, der u. a. für die emotionale Verarbeitung von Schmerzen zuständig ist [14].

### Stomatognathes System (kraniomandibuläres System).

Die sehr enge Vernetzung zwischen den spinalen Trigeminuskernen und den WDR-Neuronen der oberen drei Zervikalsegmente zeigt sich hier besonders stark ausgeprägt. Sämtliche propriozeptiven und nozizeptiven Afferenzen aus dem N. trigeminus, also die oberflächlichen wie die tiefsomatischen Afferenzen, bewirken eine direkte Konvergenzreaktion auf die entsprechenden zervikalen Efferenzen. Allerdings können umgekehrt auch die tiefsomatischen Afferenzen der Segmente C1–C3 den motorischen Trigeminuskern beeinflussen, der dann über den N. mandibularis die Kaumuskulatur ansteuert [4, 18]. Das Vorliegen einer kraniomandibulären Dysfunktion kann die Schmerzverarbeitung im Sinne einer sekundären Hyperalgesie des gesamten Organismus ungünstig beeinflussen und sollte bei rezidivierenden, chronischen Schmerzsyndromen immer mit untersucht werden [19]. Eine adäquate Therapie mit beispielsweise einer Aufbissschienenversorgung kann Einfluss auf die Körperstatik im Sinne einer lotnahen axialen Körperpositionierung haben und den muskulären Tonus vor allem kaudaler Muskelfunktionsketten reduzieren [20].

### Verkettungen nach kaudal.

Am hochzervikalen Querschnitt des Rückenmarks finden sich verzahnte Endigungsfelder der Afferenzen vor allem der Spinalnerven C2, C3 und des N. trigeminus, aber auch von C1, N. glossopharyngeus und N. facialis [21, 22]. Zudem finden sich hier deren propriozeptive Afferenzen, die unter anderem direkt zum Nucleus cervicalis centralis (CCN) projizieren. Der CCN ist Ursprungskern spinozerebellärer Projektionen, die auch zur Extremitätenmuskulatur absteigen und der Vermittlung tonischer Halsreflexe dienen. Ebenso finden sich Projektionen zu Vestibulariskerngebieten, die für die Stützmotorik von zentraler Bedeutung sind. Über die propriozeptiven Afferenzen der Spinalnerven C2/C3 und deren Projektion auf den CCN, sowie propriozeptiver Neurone, die zum unteren Zervikal- und Lumbalmark projizieren, lassen sich Verkettungen bis in die Beckenregion erklären [17].

Das komplette Spektrum des zervikozephalen Syndroms (Tab. 3) kann aus der „zervikotrigeminalen Konvergenzreaktion“ im Hirnstamm und ihren weiteren Projektionen verstanden werden. Damit wird auch nachvollziehbar, dass bei Vorliegen einer dem zervikozephalen Syndrom zugehörigen Dysfunktion der Segmente C0/C1 bis C3/4 durch entsprechende manualmedizinische Behandlung mittels segmentaler antinozizeptiver, propriozeptiver Aβ-Faser vermittelter Afferenzierung eine Beschwerdebesserung erreicht werden kann.

## Die untere Halswirbelsäule und das zervikobrachiale Schmerzsyndrom

**Figure 4:**
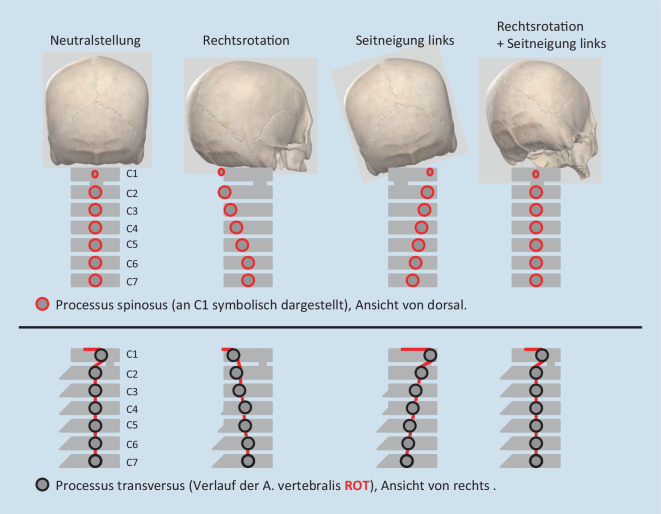

Vom 3. HWK mit dem Übergangssegment C3/C4 (C4) bis zu Th1 bzw. dem Segment C7/Th1 (C8) sprechen wir von der unteren HWS, die durch relativ vergleichbar aufgebaute Halswirbelkörper gebildet wird. Auch deren Bewegungsspektrum ist in allen drei Dimensionen ähnlich (Tab. 1). Es gilt für die HWS ab C2/3 eine gesetzmäßige Bewegungskoppelung (Fryett-Regel), die besagt, dass sich Rotation und Seitneigung der Halswirbelkörper gleichsinnig bewegen. Bei einer Seitneigung nach rechts rotieren die HWK ebenfalls nach rechts, besonders gut an der Abweichung der Dornfortsätze nach links palpabel. Diese Koppelung kann in der manuellen Medizin durch Rotation und kontralaterale Seitneigung (sogenannte 15°/15°-Regel) und der damit erreichten funktionellen Neutralstellung der HWS zum Schutz der A. vertebralis bei bestimmten manipulativen und auch mobilisierenden Techniken eingesetzt werden ([23]; Abb. 4).

Neuroanatomisch betrachtet konvergieren im Bereich untere HWS die Afferenzen der Segmente C3/C4 (Übergangssegment) bis C7/Th1, die aus allen Gewebearten des Nacken-Schulter-Arm-Bereichs nozizeptiven Input vermitteln und damit Schmerz und Dysfunktion auslösen können. Wenn der Schmerzreiz (nozizeptiver Reiz) entsprechend stark und lange persistiert, können Sensibilisierungsvorgänge ausgelöst werden, die aufgrund der multirezeptiven und segmentüberlagernden Konvergenzen am WDR (spinothalamisches Projektionsneuron) ein übertragenes Schmerzbild („referred pain“) entstehen lassen [5]. Es werden auch zuvor unbeteiligte Regionen schmerzhaft (Ausweitung des rezeptiven Schmerzfeldes, „pseudoradikulärer Schmerz“), d. h. das klinische Schmerz-Dysfunktions-Bild überschreitet die segmentanatomischen Grenzen und ist im Gegensatz zum radikulären Schmerz nicht mehr einem Dermatom, Myotom oder Sklerotom zuzuordnen [24]. Die topografische Zuordnung über den Thalamus auf den sensorischen Kortex (Gyrus postcentralis) dehnt sich im betroffenen Bereich des sensorischen Homunculus aus. Eine primäre Epikondylopathie kann sich so z. B. zu einem Nacken-Schulter-Arm-Syndrom aufbauen.

**Figure 5:**
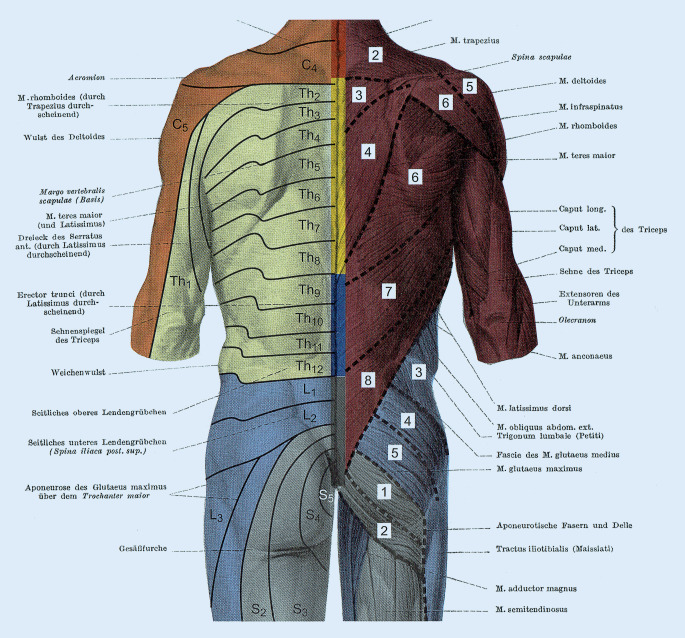

Komplex ist die Verschaltung des Plexus brachialis (C5-Th1) mit der Ausbildung von Trunci und Fasciculi, den supra- und infraklavikulären Abgängen und den verschiedenen proximalen und distalen anatomischen Engstellen und ihren verschiedenen klinischen Bildern. Den Segmentnerven C5–Th1 fehlen die ventralen Rumpfdermatome, da diese bei normaler embryonaler Entwicklung in die oberen Extremitäten ausgelagert werden. Einen Dermatomsprung, wie z. B. von C4 zu Th1 an Brust und Rücken, nennt man Hiatuslinie [25]. Zusätzlich breiten sich die Myotome der unteren HWS weit nach distal aus. Die Segmentnerven C3–C5 versorgen unter anderem als N. phrenicus motorisch und sensibel das Zwerchfell. Die rechte Schulter als Head-Zone von Leber und Gallenblase, die beispielsweise bei einer akuten Cholezystitis schmerzhaft werden kann, erklärt sich über die faszialen Verbindungen des Organs zum Zwerchfell, dessen segmentale Innervation (C3–C5) und die Konvergenz mit segmentgleichen Hautafferenzen, in diesem Fall eben seitengleich aus dem Bereich der rechten Schulter. Ebenso können über Noziafferenzen auf Segmentebene motorische Veränderungen ausgelöst werden, die eine Blockierung der HWS im Bereich HWK 3–5 auslösen können [25]. Der N. thoracodorsalis aus C6–C8 innerviert den M. latissimus dorsi mit den Ansätzen bis zu seiner Ursprungsaponeurose an der Fascia thoracolumbalis, im Bereich des Os sacrum, der Crista iliaca und der LWS. Hier überlagern sich oberflächlich die Dermatome von unterer BWS, der LWS und des Sakralbereiches mit den Myotomen von C6–8, sowie den tiefer liegenden paravertebralen Anteilen der autochthonen Rückenmuskulatur (z. B. lumbaler M. multifidus). Diese anatomische Verbindung kann, neben dem M. longissimus dorsi, ein weiterer Erklärungsansatz für die Genese einer HWS-Dysfunktion bei Funktionsstörungen im Beckenringbereich, z. B. einer Iliosakralgelenkblockierung, sein (Abb. 5).

Das autonome Nervensystem (ANS) im Bereich der oberen und unteren HWS zeigt mit der Trunkus- und variablen Ganglienbildung des Sympathikus keine direkte segmentale Ordnung. Die Neurone des Sympathikus liegen im Seitenhorn des unteren Zervikalmarks (C8) und ziehen von dort im zervikalen Grenzstrang kranialwärts zu den meist drei Halsganglien, die den Kopf- und Halsbereich vor allem über das Ganglion cervicale superius versorgen. Die peripheren sympathischen Fasern verlaufen kopfwärts mit den kranialen Arterien zu ihren Erfolgsorganen. Die eigentliche segmentale Ordnung des sympathischen Grenzstranges, sofern wir sie so nennen dürfen, stellt sich normalerweise ab C8 kaudalwärts ein (siehe Artikel zur segmentalen Dysfunktion der Brustwirbelsäule). Die parasympathischen Nervenfasern verlaufen mit den Hirnnerven HN III, VII, IX und X. Der Einfluss des ANS auf die Schmerzwahrnehmung und -verarbeitung ist aktuell noch nicht durchdrungen, allerdings lassen schon die bekannten Interaktionen des ANS mit dem neuroendokrinen und limbischen System segmentübergreifende Auswirkungen auch auf z. B. ein Blockierungsgeschehen der HWS erwarten [8, 11].

## Zusammenfassung

Die Komplexität der neuroanatomischen Verschaltung erhellt, warum die Differenzierung, welchen Ursprung ein Schmerzgeschehen haben kann, nicht immer einfach und klar ist. Schmerzbilder durch Blockierungen auf Basis der Konvergenzreaktion am WDR (Schutzreflex), myofasziale Schmerzsyndrome durch muskuläre Triggerpunkte, periphere „neuralgische“ Nervenschmerzen oder radikuläre Schmerzen sind auch bei bester Untersuchung nicht immer klar voneinander zu unterscheiden oder gehen ineinander über. Umso wichtiger ist es, in dieser sensiblen Region ein entsprechendes Verständnis für die neurophysiologischen Grundlagen der segmentalen und somatischen Dysfunktion der HWS zu besitzen. Dies kann im klinischen Alltag viele sonst schwer nachvollziehbare Symptome erklären und die Manuelle Medizin als therapeutische Optionen in den Fokus rücken.

Grundlegend gilt, dass jede potenziell bedrohliche Differenzialdiagnose vor jeglicher manualmedizinischer Behandlung ausgeschlossen sein muss. Dazu zählt vor allem der V. a. auf eine Dissektion der supraaortalen Gefäße, akute Entzündungen (z. B. Grisel-Syndrom), destruierende Prozesse (z. B. Tumoren) oder rheumatoide Arthritis (v. a. an C0/C1/C2). Die Kenntnisse der Kontraindikationen ist essenzieller Bestand jeder manualmedizinischen Ausbildung.

Ebenso gilt selbstverständlich, dass durch eine sorgfältige manualmedizinische Diagnostik eine klare Indikation zur manualmedizinischen Therapie bestehen muss. Richtungsweisend ist hier, neben der exakten Anamnese und dem orientierenden Ganzkörperstatus, die MIP-Diagnostik. Diese beinhaltet das Auffinden der segmentalen Hypomobilität, des Irritationspunkte als myofasziales Korrelat der Blockierung auf neurophysiologischer Basis des Schutzreflexes sowie die klare Feststellung der freien Richtung im Rahmen des Provokationstests. Sollte diese nicht gegeben sein, ist eine Manipulation obsolet. Besonders im Bereich der HWS sollte eine sorgfältige multidisziplinäre Abklärung erfolgen.

## Fazit für die Praxis

- Das variable klinische Bild einer funktionellen Störung im Bereich der Halswirbelsäule (HWS) lässt sich durch die komplexen Verschaltungen im Rahmen der zervikotrigeminalen und intersegmentalen Konvergenzreaktion erklären.
- Eine segmentale und somatische Dysfunktion im Bereich der Halswirbelsäule kann Ursache und/oder Symptom eines zervikozephalen Syndroms (vor allem obere HWS) oder der Zervikobrachialgie (vor allem untere HWS) sein.
- Nach Ausschluss gefährlicher Differenzialdiagnosen und spezifischer Kontraindikationen kann eine manualmedizinische Therapie durch Setzen eines antinozizeptiven propriozeptiven Reizes zu einer Beschwerdelinderung beitragen.
- Die Indikation zu einer manualmedizinischen Therapie wird aus einem klaren diagnostischen Algorithmus abgeleitet, der immer eine segmentale Mobilitätsprüfung (M), eine Irritationspunktsuche (I) und dessen Provokation (P) beinhalten sollte.
- Für manualmedizinisch tätige Ärzte eröffnet sich mit diesen Kenntnissen ein erweitertes Spektrum möglicher Indikationen für eine manualmedizinische Therapie jenseits der klassischen strukturorientierten Behandlungsindikationen.

## Abkürzungen

akt: Aktiv
ANS: Autonomes Nervensystem
ATM: Autochthone Muskulatur
CCN: Nucleus cervicalis centralis
E: Extension
F: Flexion
HN: Hirnnerven
HWK: Halswirbelkörper
HWS: Halswirbelsäule
Ink: Inklination
Ins: Insertionspunkte
IP: Irritationspunkt
LWS: Lendenwirbelsäule
MIP: Mobility, Irritation, Provocation
pas.: Passiv
R: Rotation
Rek: Reklination
ROM: „Range of motion“
SN: Seitneigung
WDR: „Wide dynamic range“
